# Establishment of the Norwegian hearing register for children

**DOI:** 10.3389/fnhum.2024.1400005

**Published:** 2024-07-29

**Authors:** Tone Stokkereit Mattsson, Ann Helen Nilsen, Siri Wennberg

**Affiliations:** Department of Medical Quality Registries, St. Olav’s University Hospital, Trondheim, Norway

**Keywords:** medical quality register, quality indicators, newborn hearing screening, hearing loss, congenital cytomegalovirus, patient reported outcome measures

## Abstract

**Introduction:**

The Norwegian Directorate of Health approved the Norwegian Hearing Register for Children in 2022. The main objective of the register is to improve the quality of treatment for children with permanent hearing loss, by measures, follow-ups and monitoring the quality and results of the health care system.

**Methods:**

Inclusion criteria are children who do not pass universal newborn hearing screening and/or children with permanent hearing loss <18 years of age. Hearing loss is defined as pure-tone audiometry threshold of (PTA4) > 20 dB in at least one ear. Data are registered at the Ear, Nose and Throat departments at inclusion and at follow-ups at the age of 3, 6, 10, and 15 years. The register collects information about the child within a holistic perspective. The key elements of the register are (a) data concerning newborn hearing screening; (b) data concerning hearing, medical information, hearing amplification and intervention (c) patient reported outcome measures registered by caregivers using three questionnaires; Pediatric Quality of Life Inventory, Strengths and Difficulties Questionnaire and Parents’ Evaluation of Aural/Oral Performance of Children.

**Results:**

The register has established four quality indicators regarding newborn hearing screening and early intervention (a) the rate of false positive neonatal screens; (b) testing for congenital cytomegalovirus within 3 weeks of age for children who do not pass newborn hearing screening; (c) audiological evaluation to confirm the hearing status no later than 3 months of age and (d) initiated intervention within 3 months after confirmation of hearing status.

**Discussion:**

The register will include the total population of hearing impaired children over long time periods. Thus, the register enables each hospital to monitor their quality indicator scores continuously and compare them with national levels in real time. This facilitates and accelerates identification of improvement areas in the hospitals and will be an important contributor for quality improvement in NHS, diagnostics and hearing intervention for children in Norway. In addition, data from the register will be a unique source for research, and study designs with a long follow-up time can be applied.

## Introduction

In many countries, there is an increasing interest in medical quality registries (MQR) as an important tool for quality improvement in health care. Primarily, MQR are intended to measure, follow-up and monitor the quality and results of the health care system at various levels. MQR provide data for disease monitoring and clinical and epidemiological research. The MQR quality indicators (QI) help to evaluate whether the health care provided meets the requirements of good quality care. MQR can also be used to induce quality improvements (Solomon et al., 1991; Mascher et al., 2017), in addition to monitoring compliance of professional guidelines and best practice. The use of patient-reported data (PROM) is required in a MQR. The overall goal of patient-reported data is to promote patient-centered treatment, where the caregivers can actively contribute to decisions about their child’s health. Further, the use of PROM help to ensure the effectiveness and relevance of the MQR (SKDE, 2024).

**Figure 1:**
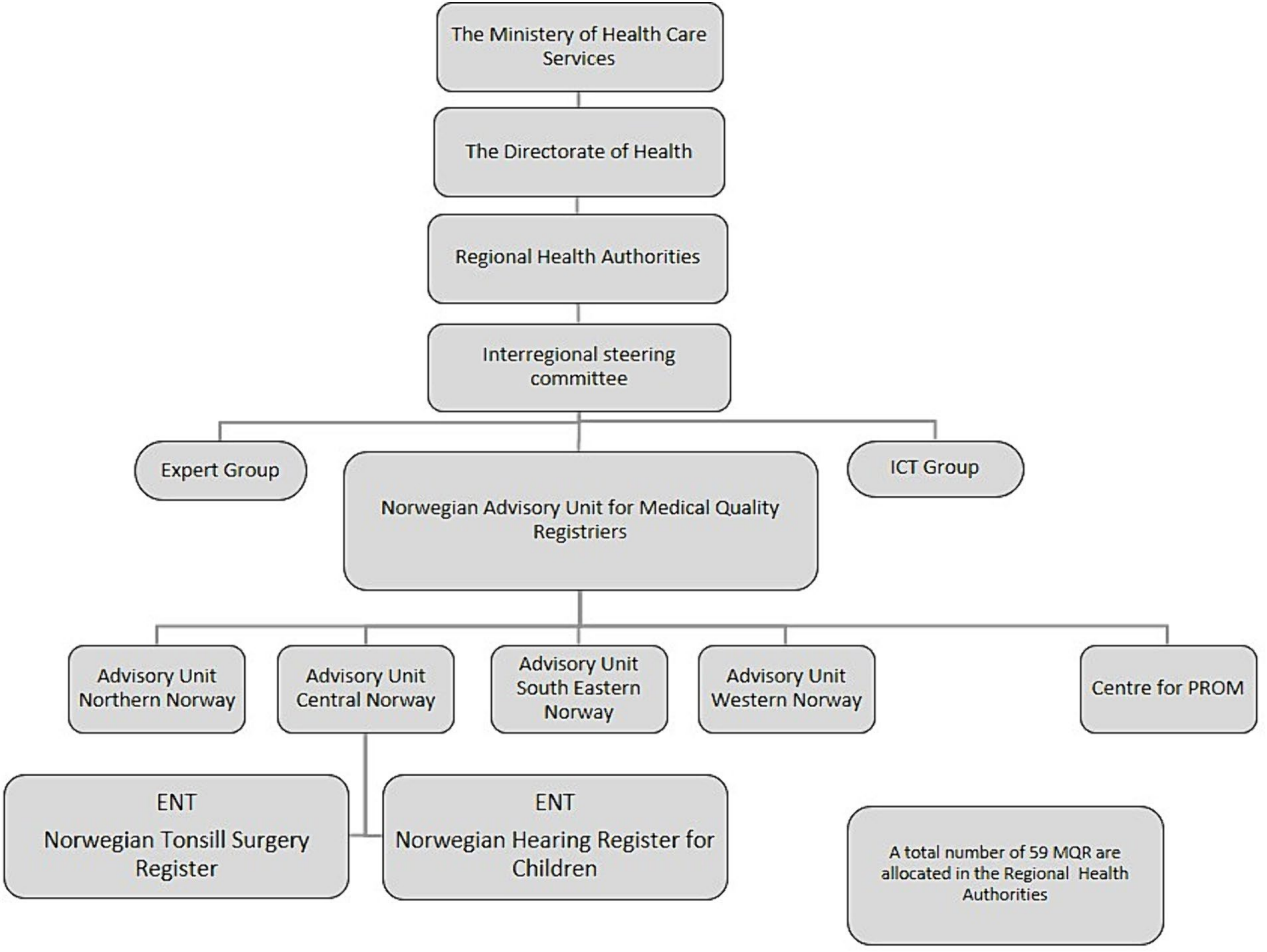
Illustrates the organization of medical quality registries in Norway. ICT, information and communication technology; ENT, ear nose and throat field; MQR, medical quality registries.

In Norway, the Regional Health Authorities have the financial and legal responsibility for the operation of the 59 MQR. The Ministry of Health and Care Services gives the health regions specific assignments for the development of quality registries such as data quality, completeness, coverage, availability of results for clinicians and patients and use in clinical improvement. The registries are supported by the Norwegian Advisory Unit for Medical Quality Registries with local offices in all four health regions (Figure 1). Each register has a steering committee, together with a register secretariat responsible for daily and professional operations (Mascher et al., 2017). The registries are subject to annual review from the national Expert group in order to ensure that the MQR function according to their purpose, and in line with requirements for operation and reporting, to maintain the status as a national quality register (SKDE, 2024).

To meet the demand from patients, health care providers and payers, the Norwegian Association for Otorhinolaryngology Head and Neck Surgery initiated the development of the Norwegian Hearing Register for Children (NHRC) in 2019. This was planned as the second MQR in the field, after the Norwegian Tonsil Surgery Register. The vision behind NHRC was to improve outcomes for every child through a high-quality screening program, safe and effective assessment and family centered early intervention (FCEI) for children with permanent hearing loss (CPHL). The register will collect information from children who fail the NHS and all CPHL within a holistically perspective and facilitate audiological research. This initiative was supported by the audiological and pediatric professional organizations and user organizations in Norway, which all participated actively in the development of the register.

The Norwegian population of 5.5 million is relatively homogenous and the healthcare system predominantly tax-funded, all which are beneficial prerequisites for establishing reliable registries (Magnussen et al., 2009; SSB, 2024). In 2023, a total of 51.980 new citizens were born in Norway (SSB, 2024).

International studies have shown that the prevalence of CPHL are 2/1,000 live births (Thompson et al., 2001; Kennedy et al., 2005; Nelson et al., 2008). The prevalence of sensorineural hearing loss (SNHL) increases with age, from 3/1,000 children at the age of 6 (Fortnum et al., 2001) and at adolescence between 3.5/1,000 bilateral and 23/1,000 cases of uni- or bilateral hearing loss (Morton and Nance, 2006; Lin et al., 2011; Korver et al., 2017). For neonates with risk factors, the prevalence increases 10-folds (Robertson et al., 2009).

It is well recognized that hearing loss may influence speech and language development, academic achievement, and social and emotional outcomes (Lieu et al., 2020). Psychosocial outcomes include emotional, social, and behavioral aspects, areas associated with one’s mental health (WHO, 2022). Quality of life in children is also affected by hearing loss (Roland et al., 2016). Early detection and targeted intervention is of uttermost importance for the child to achieve its true potential (Yoshinaga-Itano et al., 2010; Tomblin et al., 2014, 2020). We have so far no overview of the prevalence of CPHL in Norway.

Cytomegalovirus (CMV) is the most common congenital infection with a prevalence of 0.3–1.2% in industrialized countries, and the most frequent non-genetic cause of congenital SNHL in children (Fowler and Boppana, 2006; Kenneson and Cannon, 2007; Engman et al., 2008). Symptoms at birth vary, and only 10–15% will be detected on routine neonatal examinations, the majority remaining undiagnosed (asymptomatic). Long-term consequences, such as hearing loss, visual or neurological problems, are common in both symptomatic and asymptomatic children, where SNHL occurs in 15–20% overall (Barbi et al., 2003; Pass, 2005; Dollard et al., 2007; Lombardi et al., 2010; Cannon et al., 2014; Demmler-Harrison, 2016; Fowler and Boppana, 2018). CMV testing within the 21 day period after birth is required to differentiate cCMV from postnatal acquired CMV, which is not associated with childhood hearing loss (Kenneson and Cannon, 2007). The development of a rapid method for detecting CMV in saliva or urine has allowed early diagnostics with reduced costs for the society (Boppana et al., 2011).

The Universal Newborn Hearing Screening Program for Norway (NHS) was introduced across the country in a phased and nationally driven process between 2006 and 2008, and regionally implemented. A record whether the NHS is performed or not is automatically created in the Medical Birth Registry of Norway, further information is not collected (Folkehelseinstituttet, 2024).

NHS are conducted at all hospitals with a birth unit. Children who fail the NHS are directly referred to outpatient rescreen at the Ear, Nose and Throat department (ENT). Diagnostic and intervention clinics for children with hearing loss are available at a limited number of hospitals. Cochlear implantations in children are performed in Oslo University Hospital only.

In 2014 an evaluation of the program was commissioned by the Department of Health and Ministry of Education and Research. The report revealed large geographical differences in the quality of NHS and FCEI offered (Eikli et al., 2014). As a consequence, national guidelines for NHS and the habilitation of hearing impaired children (aged 0–3 years) were developed (Helsedirektoratet, 2016a,b). Screening protocols for newborns and newborns in risk were established. For babies without risk, a two-technology procedure with TEOAE (with maximum two attempts) followed by automatic brain stem response (AABR) if required was implemented. For babies in risk, AABR was implemented to detect auditory neuropathy spectrum disorder. Newborns with microtia/atresia and meningitis were defined as screen referrals, and directly referred for audiological assessment. A refer in at least one ear was considered screen referrals and followed up with outpatient rescreen with TEOAE and AABR at the local ENT department within 4 weeks of age.

The NHS program is hospital based. Babies are screened in hospitals before discharge or in outpatient screening clinics for those babies that do not complete screening before discharge. The prevalence of planned homebirths is about 1 per 1,000 births, with 100–170 occurring every year (Folkehelseinstituttet, 2024). These babies are offered NHS at scheduled outpatient follow up at local maternity institutions within 72 h after birth.

**Figure 2:**
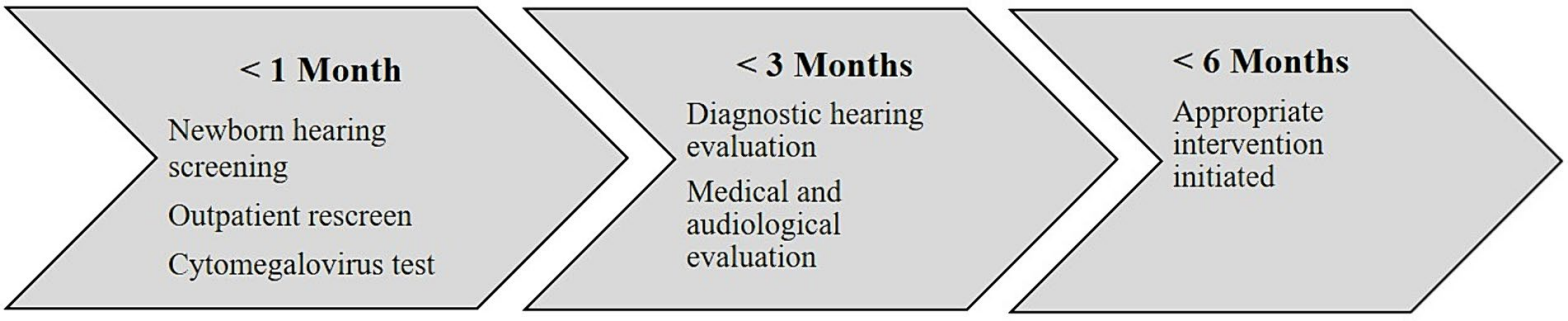
Illustrates the 1–3–6 benchmark goals for NHS and early intervention.

In addition, hearing targeted early congenital CMV screening before 3 weeks of age was implemented in newborns with referred NHS. According to the national guidelines, NHS and outpatient rescreen should be performed no later than 1 month of age. Those who fail the NHS should receive a comprehensive audiological evaluation by no later than 3 months of age and appropriate intervention with FCEI and hearing technology initiated before 6 months of age. This national 1–3–6 benchmark (JCIH, 2019) goals are important for early access to optimal language and learning, and highlight the importance of rapid identification and intervention (Figure 2). However, no national information system for monitoring the quality standards were developed, resulting in lack of information about the quality of NHS, outpatient rescreen, and audiological follow up. This inadequacy may lead to treatment based on professionals’ preferences rather than evidence-based medicine. A register will provide increased knowledge about this patient group and can improve the treatment quality for these children.

The Norwegian Directorate of Health approved the NHRC in 2022. A register secretariat and a web-based medical registration system (MRS) was established at Central Norway Regional Health Authority, St. Olav’s Hospital, Trondheim University Hospital. The register was officially launched in January 2023.

## Materials and methods

**Figure 3:**
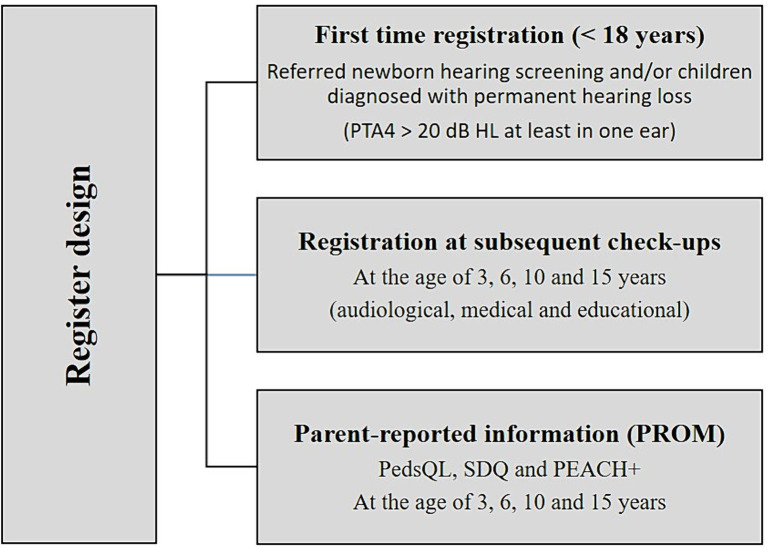
Illustrates the register design and the variety of data collected. PTA4, Pure-Tone Audiometry average over the frequencies 0.5, 1, 2 and 4 k Hz > 20 dB; Hz, Hertz; dB, decibel; PedsQL, Pediatric Quality of Life Inventory; SDQ, Strength and Difficulties Questionnaire; PEACH+, Parents Evaluation of Aural/Oral Performance of Children.

The NHRC has a prospective, longitudinal register design which includes a variety of data. The register is fully web-based, and registrations are carried out by audiological personnel and caregivers/children at several time-points (Figure 3).

### Participants

The NHRC aims to include all children in Norway who do not pass universal NHS and/or CPHL <18 years of age. Hearing loss is defined as pure-tone audiometry average over the frequencies 0.5, 1, 2, and 4 k Hz (PTA4) > 20 dB in at least one ear (Humes, 2019). In addition, CPHL diagnosed before the register was established, will be included at their audiological follow-ups. Caregivers and children receive oral and written information about the register and can decline or opt out from participation.

### Coverage

All national medical quality registries are obliged to reach a coverage of at least 80% within 5 years of operation (SKDE, 2024). Analysis for the coverage will be performed based on data from the Norwegian Patient Registry, which covers all public specialist health-care services in Norway, including private institutions and medical specialists contracted to the regional health authorities (Bakken et al., 2020).

The NHRC is in collaboration with the Swedish Hearing Register for Children developing a valid procedure for measuring the coverage rate.

### Data collection

Data are collected at the ENTs at inclusion and at follow-ups at the child’s age of 3, 6, 10, and 15 years. The web-based medical registration system (MRS) is utilized to collect data and consists of two schemes: Initial registration form and Follow-up forms. By completion, data is transferred to a national data base. Data collected are patient demographics, data from NHS, outpatient referral, diagnostic and medical hearing evaluation, intervention, and patient reported outcome measures (PROM). Specifications are listed in Table 1.

**Table 1:** Illustrate a brief description of the main variables in the NHRC.

Variables	Definition
**Initial registration form**	
Demographics	Age, gender, residency
Birth information	Gestational week, weight, CMV-test
Newborn hearing screening	Passed/referred TEOAE, AABR
Outpatient rescreening, ENT	Tympanometry, TEOAE, AABR
Diagnostic testing	Tests used to confirm hearing loss (ABR, ASSR, TEOAE, audiometry, impedance testing)
	Hearing thresholds range
	Cause of referral
Primary intervention	Initiation of hearing intervention, hearing aid/CI
	Referral early intervention program
	Referral for ophthalmologic evaluation
Follow-up forms	Hearing thresholds range
Hearing loss, hearing aid, speech	Hearing aid/CI
	Verification, compliance
	Hearing assistive technology systems
Communication	Method, use and communication level
	Family-centered early intervention
	Speech and language therapy
	Preschool/school environment
	Individualized education service
Medical and otological evaluation	Etiology; congenital or acquired, auditory neuropathy, syndromes associated with hearing loss, malformation of ear
	Genetic counseling and evaluation
	Additional disabilities other than hearing loss
PROM	Parents’ evaluation of aural/oral performance of children
	Pediatric quality of life inventory
	Strengths and difficulties questionnaire

CMV, cytomegalovirus; TEOAE, transient evoked otoacoustic emission; AABR, automated auditory brainstem response; ABR, auditory brainstem response; ASSR, automated steady-state response; CI, cochlea implants; NHRC, Norwegian hearing register for children.

### Patient reported outcome measures

PROM are electronically reported by caregivers using three questionnaires:

*Pediatric quality of life (PedsQL)* measures health-related quality of life in healthy children and adolescents and those with acute and chronic health conditions (Varni et al., 2001). Four multidimensional scales measure the core dimensions of health as delineated by the World Health Organization (WHO, 1995), as well as role (school) functioning: physical, emotional, social and school functioning. Caregivers answer questions about their child’s quality of life aspects over the past month at the NHRC’s follow-ups at age 3, 6, 10, and 15 years old.

*Strengths and difficulties questionnaire (SDQ)* is a short questionnaire that maps psychosocial functioning in children and adolescents (Goodman, 2001). It is not a diagnostic tool, but a mapping chart. The questionnaire has 25 statements about children, where caregivers should indicate whether each statement is appropriate for their child, based on the last six months. The questions are divided into five scales: emotional symptoms, conduct problems, hyperactivity/inattention, peer relationship problems, and prosocial behavior (Goodman, 1997). Caregivers answer the questionnaire at the NHRC’s follow-ups at age 6, 10, and 15 years old.

*Parents evaluation of aural/oral performance of children (PEACH+)* is used to evaluate the effectiveness of hearing aids/cochlear implants and provides an assessment of the child’s hearing and listening in noise and non-noise situations (Ching and Hill, 2007). Caregivers are asked to answer how often their child displays different types of listening behavior (e.g., recognizes the voices of familiar people). In addition, the caregivers indicate how easy or difficult they think the situation is for the child. Caregivers answer the questionnaire at the NHRC’s follow-ups when children are 3, 6, and 10 years old.

### Quality indicators

The design and variables of the NHRC covers the national guidelines for NHS and the intervention of CPHL (aged 0–3 years) (Helsedirektoratet, 2016a,b). The registers steering committee decide on quality indicators reflecting the national guidelines and best practice. The quality indicators are continuously evaluated and updated. The initial indicators were chosen to improve NHS, targeted CMV screening and early intervention, following the 1–3-6 rule.

Based on these guidelines, four quality indicators are established:

- The proportion of children with false positive results at newborn hearing screening
- The proportion of children tested for cCMV within 3 weeks of age in children with referred newborn hearing screening
- The proportion of children receiving diagnostic hearing evaluation within 3 months of age
- The proportion of children with initiated intervention within 3 months after confirmation of hearing status

The quality indicators are continuously displayed for each hospital along with national results for the last 12 months in the register web-solution MRS.

### Legal aspects and ethics

The NHRC is approved by The Norwegian Directorate of Health and operates in accordance with Norwegian legislation: the Health Data Filing System Act and the Health Personnel Act. The handling of personal data in the register are also regulated by the General Data Protection Regulation and the Personnel Data Act. Before inclusion at the ENT, the caregivers and children are informed about the NHRC in oral and written form, the longitudinal follow up until adulthood and their right to decline registration. The information includes possible future use of the data in research. If families opt out, this is noted in the web-based register solution, and further communication ceases (including PROMS).

According to Norwegian legislation, the NHRC do not need signed consent from the individual registered patient. When registered, children remain in the NHRC indefinitely with infrequent follow-ups. The caregivers have the right to know what information has been recorded, request correction and have all their data deleted from the register. All research conducted on data from the NHRC must be approved by the regional committees for medical and health research ethics and approval must be obtained before use of data. The children and their caregivers may be contacted for additional consent for specific research questions not covered by the register. If a single registration unit wants to use their own data for quality improvement, no approval is needed.

When receiving the electronic PROMs, the caregivers must give consent to each questionnaire in order to answer. Thus, it is considered ethically sound.

The NHRC was officially launched in January 2023, and by 2024, all hospitals report data to the register. Information material have been prepared such as patient information about privacy and reservation rights, user manuals for registration and patient responses, available at the registers web-page (NHRC, 2024).

## Results

Results presented in this paper are preliminary findings. All current ENT departments have started including CPHL during the first year after establishment of the register, a total of 36 units. After the first year of collecting data, we do not have a correctly estimated coverage rate. This means that, for the time being, one must be careful with the interpretation of preliminary results based on data from the register.

About 2,200 children were included during the first year of operation. This number represents 681 infants with referred NHS in 2023. The remaining number of children are diagnosed with later onset hearing loss during 2023 or previously diagnosed CPHL before the register was launched.

Due to the recent launch, we choose to present a few selected results from the NHS. A number of 681 newborns were included based of referred NHS. Of these newborns, 14% (*n* = 93) were diagnosed with congenital permanent hearing loss. Among these newborns, the distribution was 2/3 with a bilateral hearing loss and 1/3 with a unilateral hearing loss.

Of the 681 newborns with referred NHS, a CMV test was confirmed in 71% (*n* = 480) and not performed in 8%. It is unknown whether the test was performed in 21% of the newborns. The results from the confirmed CMV tests were negative in 90% (*n* = 433), unknown in 9% and positive in 1%. Of the six CMV positive newborns, two were diagnosed with a permanent hearing loss.

## Discussion

The register will provide knowledge about a population that until now has been difficult to access in Norway. Among other things, epidemiological research has been challenging, both nationally and internationally. The consequence is that knowledge about CPHL has been based on small clinical studies or deals with sub-groups within the population. The establishment of a national quality register with a whole-population sample that follows children from NHS until they are adults is unique.

Aggregation of data from other national and international registries to compile data will increase knowledge of the population. The Norwegian Neonatal Network database contains information on all patients admitted to the country’s neonatal wards, both sick children born after full-term pregnancy and children born prematurely. Aggregation of data between the two registries will provide valuable, supplementary information regarding this sub-population. Collaboration with international registries may form basis for observational and intervention research (ANCHOR, 2024; Nationellt kvalitetsregister för öron-, näs- och halssjukvård, 2024).

### Guiding values in the development of the register

In Norway, focus has been on early hearing detection and intervention to facilitate the children’s optimal outcome. The NHS, early amplification and early language interventions based on best practice protocols has been key tools to optimize communication and linguistic competence. However, the longitudinal perspective with the CPHL and their families in focus, including long-term developmental, educational, and psychosocial outcomes and health-related quality of life, has been lacking.

By designing the NHRC within a holistic perspective that follows children with all degrees of permanent hearing loss from birth to adulthood, the multi-professional team has provided an evidence-based tool to provide the best possible care for children both at present and in future. We believe that the involvement of the professional and user organizations has been a key-factor in the flying start of the NHRC, and reflects the strong demand in the professional societies for transparency and quality improvement.

When developing the register, the importance of maintaining simplicity to avoid complications and ensure effective data collection was emphasized. At the same time, it was important to focus on holistic treatment and follow-up of CPHL, which led to the register containing quite a few variables. A number of adjustments were made in connection with piloting the register before national launching. This work was carried out with the intention to help mitigate the risk of incomplete data collection.

The structure and variables are partly based on the Swedish Hearing Register for Children, which was established in the 1990s and reinvented in 2019. Main differences between the two registries are the registration of data from the NHS, CMV-testing, and PROM in Norway. National registries with uniformly defined variables, common structure and congruent methods of data collection facilitates aggregation of data, analyses and evaluating outcomes from CPHL in the Nordic countries.

Number of participants in the NHRC increases rapidly due to inclusion of children with previously established diagnose before the register was launched, included continuously at their audiological follow-ups. This solution requires great effort from the registrars in collecting information from previous medical records. However, we believe participation in NHRC will provide a structured follow-up for these children based on national guidelines. This can increase geographical equalities in intervention and improvement of the program, and provide valuable knowledge which can lead to future changes in the national NHS, diagnostics and intervention program. Due to the longitudinal design, data from the NHRC will be a unique source for research, facilitating study designs with a long follow-up time. Studies based on registries can provide useful insights into topics that for ethical or practical reasons cannot be investigated in randomized trials (Ludvigsson et al., 2015).

The main challenge with a quality register is to achieve representable results through high coverage and completeness of register data, along with high positive predictive values of the indicators included in the register. The web-based solution is designed to avert missing data and reduce loss to follow up. Selected variables are defined as compulsory to registration. An updated overview over the units` completed and future registrations are provided in real-time. Electronical reminders are automatically sent to caregivers who do not complete the PROM questionnaires. In addition, data quality procedures are implemented to reduce missing data regarding clinical registrations and reminders to caregivers. In future, the register will pursue this focus to reduce loss to follow up and ensure representative data for the population. As part of this, newsletters to the units and caregivers covering results from the NHRC are important.

However, as a newly launched register, the method for coverage rate analysis is challenging, based on the complexity of the register. In collaboration with the experienced Swedish register, work is in progress. All national medical quality registries are obliged to reach a coverage of at least 80% within 5 years of operation (SKDE, 2024). The 100% participation ENTs during the first year of operation bodes well for future high coverage.

In addition to high coverage and completeness of variables, data collected must be valid and reliable. These are prerequisites to draw reliable conclusions from data for use in quality improvement and research. In 2024 the NHRC started a data-validation study to assess inter-rater reliability and correctness, an important part of the requirements set for the national MQR.

### Quality indicators

Measuring quality is a crucial part of the shift towards value-based health care. By measuring the outcome of patient health care, quality improvement can be initiated where professionals are provided to learn from each other in addition to use in research. The quality indicators defined in the NHRC provide a continuous measure of the quality for the program and can be used to monitor clinical practices. There is also a need to inform decision makers, healthcare providers and the public about the quality of care.

Results from the quality indicators are presented in a national web-site (SKDE, 2024). QI presented in the MRS enable each hospital to monitor their scores continuously and compare them with national levels in real time. This facilitates and accelerates identification of improvement areas in the hospitals and will be an important contributor for quality improvement in NHS, diagnostics and audiological intervention for Norwegian children. In addition, the QI can also be used to illustrate the effect of local quality projects.

However, completeness of variables may vary, both between different ENTs and between different variables in the register. This may influence the validity of the results on the QI, and whether they are representative for the population. Therefore, it is of paramount importance to have procedures which ensure validated data and high completeness in the register, as previously shown in a validation study from the Norwegian Tonsil Surgery Register (Wennberg et al., 2024).

In addition, there are plans for future data reports to meet the different requirements of the users. Preparations for research studies are made, where data from the register will be an important source. Data from MQR with relevant and reliable results are used more and more in research and as a basis for forming public health policy.

### Newborn hearing screening

An evaluation in 2017, revealed geographical differences in complying with the national guidelines for NHS and need for quality improvement (Mattsson, 2017). The Norwegian healthcare system is available to all residents. However, due to the elongated country with sparsely populated areas, many residents have long traveling distances to medical or educational services. Although NHS is conducted at all hospitals at birth unit, the limited availability of diagnostic and intervention clinics for children with hearing loss pose challenges.

Based on the annual birth rate and a well-functioning screening program, the anticipated referral rate of NHS is about 3–4% (Wood et al., 2015). Due to the gradual start of the register in 2023, a valid referral rate is not yet provided. Of the 681 children with referred NHS, 86% pass the follow-up tests. High rates of false-positive neonatal screens for CPHL may lead to unnecessary parental worry, affect the interaction between caregivers and children in a vulnerable phase and increase costs for the government (Kennedy et al., 2000). The NHRC will identify the rate of false-positive neonatal screens. Exploring key factors in well-functioning local screening units are useful and can be implemented in quality improvement projects in units with unsatisfactory rates of false-positive neonatal screens.

### Congenital cytomegalovirus

It is well recognized that children with cCMV infection have increased risk for delayed onset, fluctuating and progressive SNHL, with higher incidence in symptomatic children (Fowler et al., 1997). However, the prevalence and negative impact for the Norwegian pediatric population still remain unclear. By implementation of the NHRC results of the hearing targeted CMV screening in Norway are documented. Early identification of cCMV provides opportunities for focused surveillance and early FCEI to optimize the child’s development and language in case of hearing loss (Cannon et al., 2014; Demmler-Harrison, 2016).

In the Nordic countries, Norway is the first to implement hearing targeted early CMV screening. The preliminary results from the NHRC shows that the CMV test was performed in 71% of the children who did not pass the initial hearing screen. It is feasible for the hearing screeners to tests for CMV from saliva or urine in referred newborns. However, results indicates that despite national guidelines, hearing targeted CMV screening in newborns are still not fully implemented in clinical practice. In 21% of the newborns, the ENTs lacked information whether the test was performed. This may reflect defaults in informational float between screeners and ENTs. According to preliminary data, in 8% of the newborns the CMV test was not performed in connection with refer on the NHS. Together with the proportion of unknowns, this high number indicates a need for quality improvement. There is probably great potential in improving local routines for how the tests are performed, documented and retrieved by screeners, audiologists and doctors.

In order to improve the existing low rate of targeted CMV screening, the NHRC will initiate a quality improvement project. In 2025, all neonatal and ENT units will be invited to participate, areas of improvement identified and action initiated. Data before and after initiation will be measured and the results directly informed to clinical practice and presented on national conferences. In addition, focus on the national web-site displaying the quality indicators provide a vehicle to monitor, bring awareness to and improve the rate of targeted CMV screening.

There is evidence of antiviral therapy leading to improved hearing and neurocognitive prognosis in newborns with symptomatic cCMV (Kimberlin et al., 2015; Korndewal et al., 2015). However, data supporting the treatment of asymptomatic infants with antivirals is lacking. Early diagnosis provides opportunities for audiological follow up to identify late onset hearing loss, however the effect of FCEI in normal hearing children are not documented. In total, existing evidence is scarce and often based on small, heterogeneous groups with limited follow-up time. Data from NHRC will in future contribute to research on this field providing a unique source for longitudinal research on children with cCMV related hearing loss. Data from the register will provide knowledge about the effectiveness of a hearing targeted early cCMV approach and contribute to the decisions of policy makers in a potentially future universal cCMV screening.

Children with cCMV may pass newborn hearing screening because almost half present with delayed onset of hearing loss. Those with residual hearing are at significant risk for progression and, therefore, require careful audiologic monitoring.

### Children with mild hearing loss

Children with mild hearing loss may not be identified through NHS due to limitations of the test equipment or testing methodology used, but can later be diagnosed with permanent hearing loss. These children risk not receiving timely FCEI and remain underserved, and are as a group less frequently subject to research. Laugen has shown early detection of the hearing loss predicts better psychosocial outcomes, whereas degree of hearing loss does not affect psychosocial outcomes. FCEI is crucial for all degrees of hearing loss, including milder cases. Addressing hearing loss early can greatly impact a child’s overall well-being and future success (Laugen et al., 2016). Language outcomes for moderate to severe hearing loss has improved with timely FCEI, while this is not the case for mild hearing loss (Carew et al., 2018). Screening and intervention services must recognize the significance of early action for all degrees of hearing loss, both for language development and psychosocial development. The NHRS focus on this group of children, reflected in the hearing threshold level (PTA4 > 20 dB HL), the longitudinal register design and the holistic perspective.

### Patient reported outcome measures

The assessment of outcomes based on the patient’s perspective are increasingly accompanying the traditional clinical ways of measuring health and the effects of treatment. Quality registries including PROM can identify areas requiring improvement and evaluate the effects of changes in practice. Results from the Norwegian Tonsil Surgery Register has contributed to a change in clinical practice regarding tonsil surgery and improved patient treatment (Bugten et al., 2022).

PROM are valuable tools in NHRC for assessing children’s functioning, as they provide a holistic supplement beyond the audiological perspective. Parents have intimate knowledge of their child’s behavior and experiences, offering insights that may not be apparent during a brief clinical evaluation. This approach enhances the understanding of the child’s overall development and aids in tailoring interventions to their specific needs.

Using PROM may be helpful in evaluation of outcome in different patient populations in NHRC. Information from caregivers in clinical consultations lay the ground for structured communication for each child and enhance higher levels of family involvement. On the other side, collecting sensitive information about quality of life and psychosocial functioning in NHRC may and has been debated. In the development of the register, caregivers emphasized the choice of PROM questionnaires and the importance of assessing the child in a broad perspective. In addition, including PROM in the NHRC introduces a new, standardized tool for the audiological professionals.

### Future perspective

The vision is that the NHRC will be used in an active way as metrics for continuous learning, quality assurance, improvement, benchmarking and research to create the best health care together with the individual child.

Moreover, by utilizing data from quality registries, healthcare stakeholders can make informed decisions regarding resource allocation, policy development, and prioritization of healthcare initiatives, ultimately contributing to the shift towards value-based healthcare (McNeil et al., 2010; Larsson et al., 2012).

The NHRC will be an important source for monitoring of children with hearing loss in Norway, contributing to increased knowledge about the population within a holistic perspective. We believe that the register can contribute to a more structured follow-up of children with hearing loss, with an awareness of the treatment situation based on national professional guidelines. We strive to future, the range of data collected within a whole-population sampling frame.

## Data availability statement

The raw data supporting the conclusions of this article will be made available by the authors, without undue reservation.

## Author contributions

TM: Writing – original draft, Writing – review & editing. AN: Writing – original draft, Writing – review & editing. SW: Writing – original draft, Writing – review & editing.

## References

[ref1] ANCHOR (2024). Australian national child hearing health outcomes registry. Available at: https://www.mcri.edu.au/research/projects/anchor (Accessed May 3, 2024).

[ref2] Bakken I. J. Ariansen A. M. S. Knudsen G. P. Johansen K. I. Vollset S. E. (2020). The Norwegian patient registry and the Norwegian registry for primary health care: research potential of two nationwide health-care registries. Scand. J. Public Health 48, 49–55. doi: 10.1177/1403494819859737, PMID: 31288711

[ref3] Barbi M. Binda S. Caroppo S. Ambrosetti U. Corbetta C. Sergi P. (2003). A wider role for congenital cytomegalovirus infection in sensorineural hearing loss. Pediatr. Infect. Dis. J. 22, 39–42. doi: 10.1097/00006454-200301000-00012, PMID: 12544407

[ref4] Boppana S. B. Ross S. A. Shimamura M. Palmer A. L. Ahmed A. Michaels M. G.. (2011). Saliva polymerase-chain-reaction assay for cytomegalovirus screening in newborns. N. Engl. J. Med. 364, 2111–2118. doi: 10.1056/NEJMoa1006561, PMID: 21631323 PMC3153859

[ref5] Bugten V. Wennberg S. Amundsen M. F. Blindheimsvik M. A. B. (2022). Reducing post-tonsillectomy haemorrhage: a multicentre quality improvement programme incorporating video-based cold technique instruction. BMJ Open Qual. 11:e001887. doi: 10.1136/bmjoq-2022-001887, PMID: 36410782 PMC9680151

[ref6] Cannon M. J. Griffiths P. D. Aston V. Rawlinson W. D. (2014). Universal newborn screening for congenital CMV infection: what is the evidence of potential benefit? Rev. Med. Virol. 24, 291–307. doi: 10.1002/rmv.1790, PMID: 24760655 PMC4494732

[ref7] Carew P. Mensah F. K. Rance G. Flynn T. Poulakis Z. Wake M. (2018). Mild-moderate congenital hearing loss: secular trends in outcomes across four systems of detection. Child Care Health Dev. 44, 71–82. doi: 10.1111/cch.12477, PMID: 28612343

[ref8] Ching T. Y. Hill M. (2007). The Parents' evaluation of aural/Oral performance of children (PEACH) scale: normative data. J. Am. Acad. Audiol. 18, 220–235. doi: 10.3766/jaaa.18.3.4, PMID: 17479615

[ref9] Demmler-Harrison G. J. (2016). Congenital cytomegalovirus infection: the elephant in our living room. JAMA Pediatr. 170, 1142–1144. doi: 10.1001/jamapediatrics.2016.2892 27723888

[ref10] Dollard S. C. Grosse S. D. Ross D. S. (2007). New estimates of the prevalence of neurological and sensory sequelae and mortality associated with congenital cytomegalovirus infection. Rev. Med. Virol. 17, 355–363. doi: 10.1002/rmv.544 17542052

[ref11] Eikli G. Buan M. Fossum A. T. Landsvik B. Norup L. Stensbøl J.. (2014). CI - og hva så? Rapport fra tverrfaglig utvalg for en samordnet pedagogisk oppfølging av barn med cochleaimplantat. Available at: https://www.statped.no/globalassets/fagomrader/horsel/horsel-2/dokumenter/ci---og-hva-sa---rapport-5-2-14.pdf (Accessed February 24, 2024)

[ref12] Engman M. L. Malm G. Engstrom L. Petersson K. Karltorp E. Tear Fahnehjelm K.. (2008). Congenital CMV infection: prevalence in newborns and the impact on hearing deficit. Scand. J. Infect. Dis. 40, 935–942. doi: 10.1080/00365540802308431 18720260

[ref13] Folkehelseinstituttet (2024). Medical birth registry of Norway. Available at: http://statistikkbank.fhi.no/mfr/ (Accessed February 24, 2024).

[ref14] Fortnum H. M. Summerfield A. Q. Marshall D. H. Davis A. C. Bamford J. M. (2001). Prevalence of permanent childhood hearing impairment in the United Kingdom and implications for universal neonatal hearing screening: questionnaire based ascertainment study. BMJ 323, 536–540. doi: 10.1136/bmj.323.7312.536 11546698 PMC48157

[ref15] Fowler K. B. Boppana S. B. (2006). Congenital cytomegalovirus (CMV) infection and hearing deficit. J. Clin. Virol. 35, 226–231. doi: 10.1016/j.jcv.2005.09.016, PMID: 16386462

[ref16] Fowler K. B. Boppana S. B. (2018). Congenital cytomegalovirus infection. Semin. Perinatol. 42, 149–154. doi: 10.1053/j.semperi.2018.02.002 29503048

[ref17] Fowler K. B. McCollister F. P. Dahle A. J. Boppana S. Britt W. J. Pass R. F. (1997). Progressive and fluctuating sensorineural hearing loss in children with asymptomatic congenital cytomegalovirus infection. J. Pediatr. 130, 624–630. doi: 10.1016/s0022-3476(97)70248-8 9108862

[ref18] Goodman R. (1997). The strengths and difficulties questionnaire: a research note. J. Child Psychol. Psychiatry 38, 581–586. doi: 10.1111/j.1469-7610.1997.tb01545.x 9255702

[ref19] Goodman R. (2001). Psychometric properties of the strengths and difficulties questionnaire. J. Am. Acad. Child Adolesc. Psychiatry 40, 1337–1345. doi: 10.1097/00004583-200111000-00015 11699809

[ref20] Helsedirektoratet (2016a). Nasjonal faglig retningslinje for screening av hørsel hos nyfødte. Oslo: Helsedirektoratet.

[ref9001] Helsedirektoratet (2016b). Nasjonal faglig retningslinje for utredning og oppfølging av hørsel hos små barn (0–3 år). Oslo: Helsedirektoratet.

[ref21] Humes L. E. (2019). The World Health Organization's hearing-impairment grading system: an evaluation for unaided communication in age-related hearing loss. Int. J. Audiol. 58, 12–20. doi: 10.1080/14992027.2018.1518598, PMID: 30318941 PMC6351193

[ref22] JCIH (2019). Year 2019 position statement: principles and guidelines for early hearing detection and intervention programs. J. Early Hearing Detect. Intervention 4, 1–44. doi: 10.15142/fptk-b748

[ref23] Kennedy C. Kimm L. Thornton R. Davis A. (2000). False positives in universal neonatal screening for permanent childhood hearing impairment. Lancet 356, 1903–1904. doi: 10.1016/s0140-6736(00)03267-0, PMID: 11130392

[ref24] Kennedy C. McCann D. Campbell M. J. Kimm L. Thornton R. (2005). Universal newborn screening for permanent childhood hearing impairment: an 8-year follow-up of a controlled trial. Lancet 366, 660–662. doi: 10.1016/S0140-6736(05)67138-3, PMID: 16112302

[ref25] Kenneson A. Cannon M. J. (2007). Review and meta-analysis of the epidemiology of congenital cytomegalovirus (CMV) infection. Rev. Med. Virol. 17, 253–276. doi: 10.1002/rmv.535 17579921

[ref26] Kimberlin D. W. Jester P. M. Sánchez P. J. Ahmed A. Arav-Boger R. Michaels M. G.. (2015). Valganciclovir for symptomatic congenital cytomegalovirus disease. N. Engl. J. Med. 372, 933–943. doi: 10.1056/NEJMoa1404599 25738669 PMC4401811

[ref27] Korndewal M. J. de Vries J. J. de Melker H. E. (2015). Valganciclovir for congenital cytomegalovirus. N. Engl. J. Med. 372, 2462–2463. doi: 10.1056/NEJMc1504937 26083218

[ref28] Korver A. M. Smith R. J. Van Camp G. Schleiss M. R. Bitner-Glindzicz M. A. Lustig L. R.. (2017). Congenital hearing loss. Nat. Rev. Dis. Primers 3:16094. doi: 10.1038/nrdp.2016.94 28079113 PMC5675031

[ref29] Larsson S. Lawyer P. Garellick G. Lindahl B. Lundström M. (2012). Use of 13 disease registries in 5 countries demonstrates the potential to use outcome data to improve health care's value. Health Aff. 31, 220–227. doi: 10.1377/hlthaff.2011.0762 22155485

[ref30] Laugen N. J. Jacobsen K. H. Rieffe C. Wichstrøm L. (2016). Predictors of psychosocial outcomes in hard-of-hearing preschool children. J. Deaf. Stud. Deaf. Educ. 21, 259–267. doi: 10.1093/deafed/enw005, PMID: 26921612

[ref31] Lieu J. E. C. Kenna M. Anne S. Davidson L. (2020). Hearing loss in children: a review. JAMA 324, 2195–2205. doi: 10.1001/jama.2020.17647 33258894

[ref32] Lin F. R. Niparko J. K. Ferrucci L. (2011). Hearing loss prevalence in the United States. Arch. Intern. Med. 171, 1851–1852. doi: 10.1001/archinternmed.2011.506 22083573 PMC3564588

[ref33] Lombardi G. Garofoli F. Stronati M. (2010). Congenital cytomegalovirus infection: treatment, sequelae and follow-up. J. Matern. Fetal Neonatal Med. 23, 45–48. doi: 10.3109/14767058.2010.506753 20807160

[ref34] Ludvigsson J. F. Håberg S. E. Knudsen G. P. Lafolie P. Zoega H. Sarkkola C.. (2015). Ethical aspects of registry-based research in the Nordic countries. Clin. Epidemiol. 7, 491–508. doi: 10.2147/clep.S90589, PMID: 26648756 PMC4664438

[ref35] Magnussen J. Saltman R. B. Vrangbæk K. European Observatory on Health Services and Policies (2009). Nordic health care system: Recent reforms and current policy challenges. Maidenhead: Open University Press.

[ref36] Mascher K.L. Ekman G.J. Bartels P.D. Borre M. Vuori A. Häkinen U.. (2017). Guide for international research on patient quality registries in the nordic countries. Available at: https://www.kvalitetsregistre.no/sites/default/files/nordicguidancedocument_quality_registries_final_for_web.pdf.

[ref37] Mattsson T. S. (2017). “Nyfødtscreening av hørsel og CMV diagnostikk- 1 år etter!” in Høstmøtet, Norsk forening for otorhinolaryngologi, hode- og halskirurgi (NOLF) (Norway: Oslo).

[ref38] McNeil J. J. Evans S. M. Johnson N. P. Cameron P. A. (2010). Clinical-quality registries: their role in quality improvement. Med. J. Aust. 192, 244–245. doi: 10.5694/j.1326-5377.2010.tb03499.x, PMID: 20201755

[ref39] Morton C. C. Nance W. E. (2006). Newborn hearing screening--a silent revolution. N. Engl. J. Med. 354, 2151–2164. doi: 10.1056/NEJMra050700 16707752

[ref40] Nationellt kvalitetsregister för öron-, näs- och halssjukvård (2024). Registret för hörselnedsättning hos barn. Available at: https://hnsb.registercentrum.se/ (Accessed May 3, 2024).

[ref41] Nelson H. D. Bougatsos C. Nygren P. Force U. S. P. S. T. (2008). Universal newborn hearing screening: systematic review to update the 2001 US preventive services task Force recommendation. Pediatrics 122, e266–e276. doi: 10.1542/peds.2007-1422 18595973

[ref42] NHRC (2024). Norwegian hearing register for children. Available at: https://www.stolav.no/fag-og-forskning/medisinske-kvalitetsregistre/horselsregisteret/ (Accessed March 11, 2024).

[ref43] Pass R. F. (2005). Congenital cytomegalovirus infection and hearing loss. Herpes 12, 50–55, PMID: 16209862

[ref44] Robertson C. M. Howarth T. M. Bork D. L. Dinu I. A. (2009). Permanent bilateral sensory and neural hearing loss of children after neonatal intensive care because of extreme prematurity: a thirty-year study. Pediatrics 123, e797–e807. doi: 10.1542/peds.2008-2531, PMID: 19403472

[ref45] Roland L. Fischer C. Tran K. Rachakonda T. Kallogjeri D. Lieu J. E. (2016). Quality of life in children with hearing impairment: systematic review and Meta-analysis. Otolaryngol. Head Neck Surg. 155, 208–219. doi: 10.1177/0194599816640485 27118820 PMC5293136

[ref46] SKDE (2024). Nasjonalt servicemiljø for medisinske kvalitetsregistre. Available online at: https://www.kvalitetsregistre.no/ (Accessed February 22, 2024).

[ref47] Solomon D. J. Henry R. C. Hogan J. G. Van Amburg G. H. Taylor J. (1991). Evaluation and implementation of public health registries. Public Health Rep. 106, 142–150, PMID: 1902306 PMC1580226

[ref48] SSB (2024). The national statistical institute of Norway. Available at: https://www.ssb.no/en/befolkning/folketall/statistikk/befolkning (Accessed March 7, 2024).

[ref49] Thompson D. C. McPhillips H. Davis R. L. Lieu T. L. Homer C. J. Helfand M. (2001). Universal newborn hearing screening: summary of evidence. JAMA 286, 2000–2010. doi: 10.1001/jama.286.16.2000 11667937

[ref50] Tomblin J. B. Oleson J. Ambrose S. E. Walker E. A. McCreery R. W. Moeller M. P. (2020). Aided hearing moderates the academic outcomes of children with mild to severe hearing loss. Ear Hear. 41, 775–789. doi: 10.1097/AUD.0000000000000823, PMID: 32032223 PMC7546580

[ref51] Tomblin J. B. Oleson J. J. Ambrose S. E. Walker E. Moeller M. P. (2014). The influence of hearing aids on the speech and language development of children with hearing loss. JAMA Otolaryngol. Head Neck Surg. 140, 403–409. doi: 10.1001/jamaoto.2014.267, PMID: 24700303 PMC4066968

[ref52] Varni J. W. Seid M. Kurtin P. S. (2001). PedsQL 4.0: reliability and validity of the pediatric quality of life inventory version 4.0 generic core scales in healthy and patient populations. Med. Care 39, 800–812. doi: 10.1097/00005650-200108000-00006, PMID: 11468499

[ref53] Wennberg S. Amundsen M. F. Bugten V. (2024). A validation study of the 30-day questionnaire in the national Norwegian tonsil surgery register: can we trust the data reported by the patients? Eur. Arch. Otorrinolaringol. 281, 977–984. doi: 10.1007/s00405-023-08306-0, PMID: 37910209 PMC10796416

[ref54] WHO (1995). The World Health Organization quality of life assessment (WHOQOL): position paper from the World Health Organization. Soc. Sci. Med. 41, 1403–1409. doi: 10.1016/0277-9536(95)00112-k, PMID: 8560308

[ref55] WHO (2022). Mental health. WHO. Available at: https://www.who.int/news-room/fact-sheets/detail/mental-health-strengthening-our-response (Accessed April 15, 2024).

[ref56] Wood S. A. Sutton G. J. Davis A. C. (2015). Performance and characteristics of the newborn hearing screening programme in England: the first seven years. Int. J. Audiol. 54, 353–358. doi: 10.3109/14992027.2014.989548, PMID: 25766652 PMC4487563

[ref57] Yoshinaga-Itano C. Baca R. L. Sedey A. L. (2010). Describing the trajectory of language development in the presence of severe-to-profound hearing loss: a closer look at children with cochlear implants versus hearing aids. Otol. Neurotol. 31, 1268–1274. doi: 10.1097/MAO.0b013e3181f1ce07 20818291 10.1097/MAO.0b013e3181f1ce07 PMC3014847

